# Supplementary material for: Interrelations between Patients’ Clinicopathological Characteristics and Their Association with Response to Immunotherapy in a Real-World Cohort of NSCLC Patients
Source: Cancers (Basel). 2021 Jun 29;13(13):3249. doi: 10.3390/cancers13133249 (PMC8268100; doi:10.3390/cancers13133249)
Supplement: Supplementary file 1 [file cancers-13-03249-s001.zip › cancers-1235756-supplementary.pdf]

cancers

Supplementary Materials: Interrelations between Patients' Clinicopathological Characteristics and Their Association with Response to Immunotherapy in a Real-World Cohort of NSCLC Patients

Ana Callejo, Joan Frigola, Patricia Iranzo, Caterina Carbonell, Nely Diaz, David Marmolejo, Juan David Assaf, Susana Cedrés, Alex Martinez-Marti, Alejandro Navarro, Nuria Pardo, Ramon Amat and Enriqueta Felip

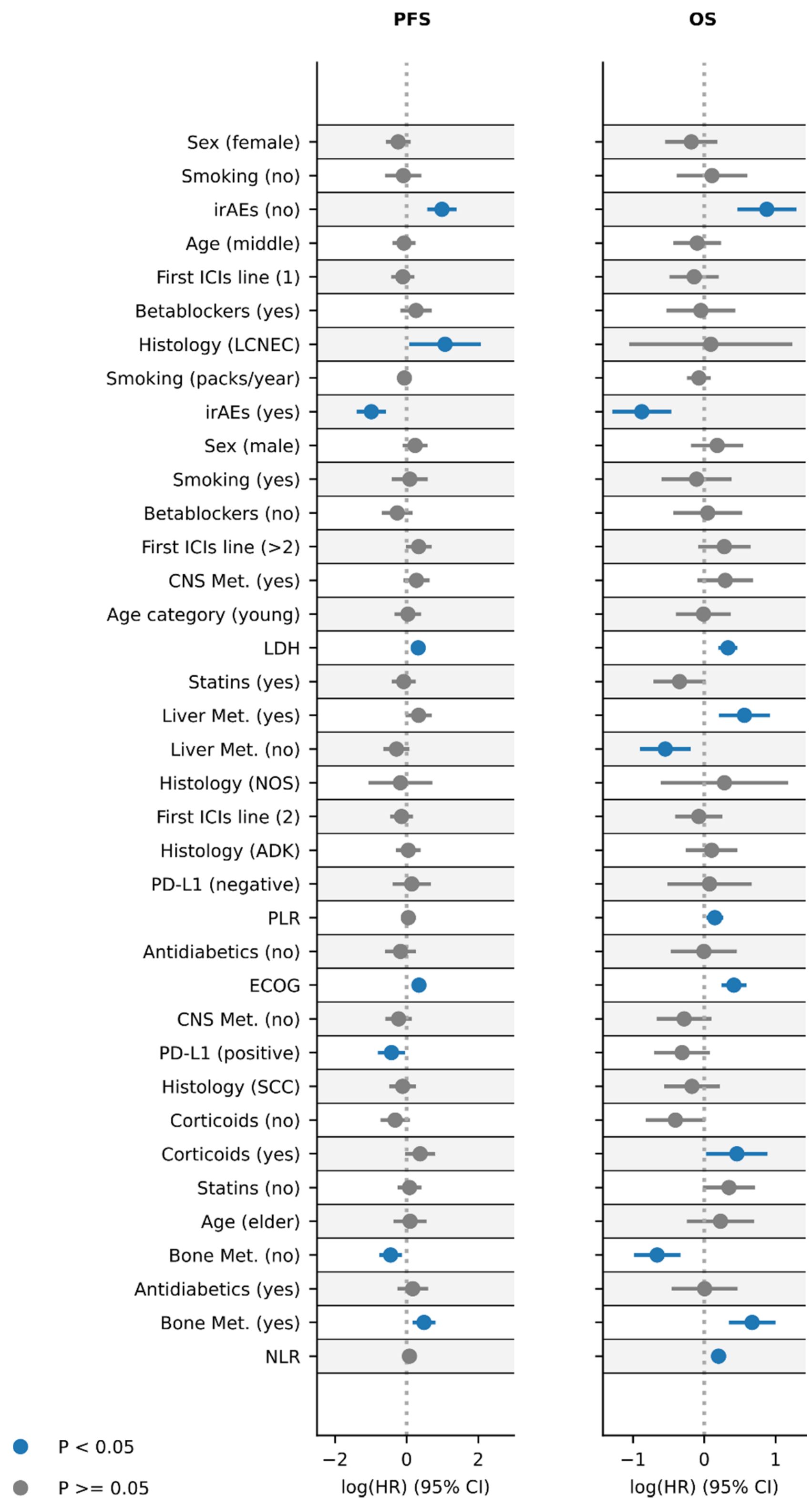

Supplementary Figure S1. Clinicopathological features associate with ICIs response in univariate survival models.

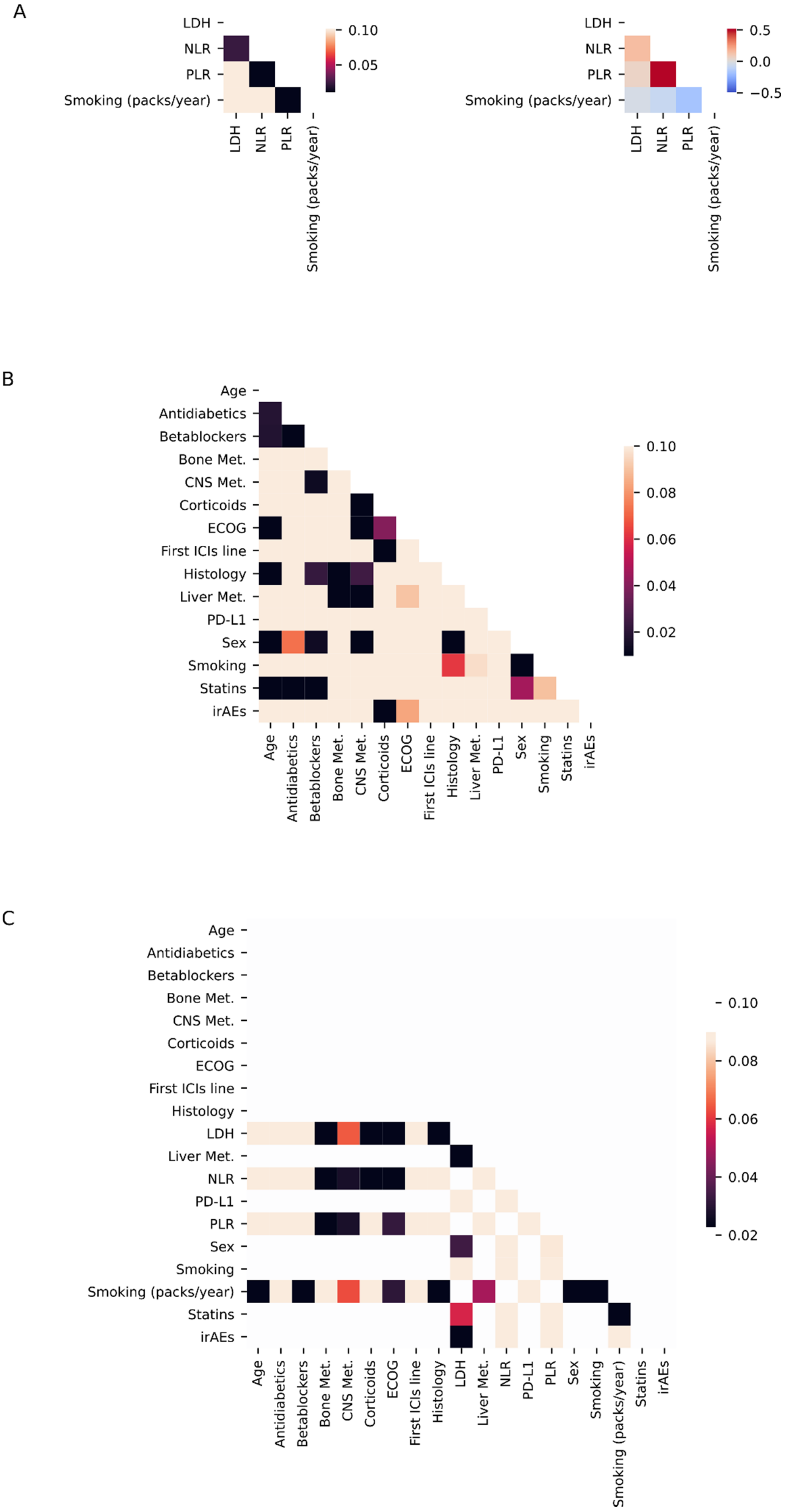

Supplementary Figure S2. Interrelations between clinicopathological features.

Supplementary Table S1. Univariate Cox proportional-hazards revival model—PFS.

Copyright: © 2021 by the authors. Licensee MDPI, Basel, Switzerland. This article is an open access article distributed under the terms and conditions of the Creative Commons Attribution (CC BY) license (http://creativecommons.org/licenses/by/4.0/).

	coef	exp(coef)	coef lower 95%	coef upper 95%	exp(coef) lower 95%	exp(coef) upper 95%	*p*
Neutrophils to lymphocytes ratio	0.078892068	1.082087524	-0.028022735	0.18580687	0.97236626	1.204189673	0.148106889
Bone Met. At ICIs start (yes)	0.48623118	1.626175891	0.166706741	0.805755618	1.181407756	2.238387226	0.002858638
Antidiabetic drugs (yes)	0.170158506	1.185492745	-0.259181586	0.599498599	0.771682883	1.821205417	0.437286177
Bone Met. At ICIs start (no)	-0.447099719	0.639480137	-0.766409864	-0.127789574	0.464678336	0.880038542	0.006063054
Age (elder)	0.097213682	1.102095847	-0.362592359	0.557019724	0.695870041	1.74546278	0.678594418
Statins (no)	0.081464148	1.084874322	-0.251239457	0.414167752	0.777836091	1.513110933	0.631293508
Corticoids pre-ICIs (yes)	0.377662241	1.458870114	-0.043330217	0.7986547	0.957595123	2.222548922	0.078707243
Corticoids pre-ICIs (no)	-0.320418794	0.725844994	-0.728776469	0.087938881	0.482498981	1.091921383	0.124075446
Histology (SCC)	-0.111103918	0.894845754	-0.482399858	0.260192023	0.61730018	1.297179151	0.557549318
PD-L1 (positive)	-0.422308117	0.655532028	-0.801197163	-0.04341907	0.448791366	0.957510042	0.028920573
CNS Met. At ICIs start (no)	-0.223582419	0.799648983	-0.590951225	0.143786387	0.553800245	1.154637436	0.232930673
ECOG at ICIs start	0.344598522	1.411423151	0.175328677	0.513868368	1.191637817	1.671745629	6.60502E-05
Antidiabetic drugs (no)	-0.170158506	0.843531101	-0.599498599	0.259181586	0.54908688	1.295869095	0.437286177
Platelets to lymphocytes ratio	0.046320561	1.047410116	-0.070638032	0.163279154	0.931799113	1.17736531	0.437613886
PD-L1 (negative)	0.143451132	1.154250404	-0.389902805	0.676805069	0.677122684	1.967581393	0.598087443
Histology (ADK)	0.045938661	1.047010187	-0.298933388	0.390810711	0.741608808	1.478178684	0.794033213
First ICIs line (2)	-0.140811425	0.868653102	-0.459730637	0.178107787	0.631453713	1.194954115	0.386831951
Histology (NOS)	-0.172476403	0.841578147	-1.064683775	0.719730969	0.344836884	2.053880578	0.70477031
Liver Met. At ICIs start (no)	-0.283072472	0.753465181	-0.648005852	0.081860909	0.523087852	1.085304843	0.128433094
Liver Met. At ICIs start (yes)	0.334748227	1.397588466	-0.030253504	0.699749958	0.970199553	2.013249247	0.072254602
Statins (yes)	-0.081464148	0.921765757	-0.414167752	0.251239457	0.660890076	1.285617897	0.631293508
LDH before ICIs start	0.32339638	1.381812966	0.18954927	0.45724349	1.208704676	1.579713481	2.18417E-06
Age category (young)	0.036367138	1.037036512	-0.335908424	0.408642699	0.714688551	1.504773967	0.848160164
CNS Met. At ICIs start (yes)	0.274390316	1.315728252	-0.093144268	0.6419249	0.911062054	1.900134931	0.143398867
First ICIs line (>2)	0.336942596	1.400658658	-0.020134523	0.694019715	0.980066823	2.00174583	0.064393235
Betablockers (no)	-0.262533297	0.769100755	-0.696656738	0.171590144	0.498248297	1.187191156	0.235908603
Smoking (yes)	0.090783419	1.095031817	-0.412357815	0.593924654	0.662087329	1.811082358	0.723606658
Sex (male)	0.23694655	1.267373374	-0.110162939	0.584056038	0.895688181	1.793297381	0.18092066

irAEs (yes)	-0.986441406	0.372901337	-1.396255385	-0.576627427	0.247522106	0.561789852	2.38497E-06
Smoking (packs/year)	-0.061603593	0.940255537	-0.218534851	0.095327665	0.803695469	1.100019234	0.441663779
Histology (LCNEC)	1.073343939	2.92514467	0.072397581	2.074290298	1.075082691	7.958896008	0.035577243
Betablockers (yes)	0.262533297	1.30021976	-0.171590144	0.696656738	0.842324334	2.007031445	0.235908603
First ICIs line (1)	-0.104945982	0.900373157	-0.427918788	0.218026823	0.651864352	1.243620425	0.524212238
Age (middle)	-0.074477831	0.928228051	-0.398030944	0.249075281	0.671641244	1.282838603	0.651875027
irAEs (no)	0.986441406	2.681674482	0.576627427	1.396255385	1.780025032	4.040043199	2.38497E-06
Smoking (no)	-0.090783419	0.913215474	-0.593924654	0.412357815	0.552156005	1.510374775	0.723606658
Sex (female)	-0.23694655	0.789033461	-0.584056038	0.110162939	0.557631997	1.11645997	0.18092066

Supplementary Table S2. Univariate Cox proportional-hazards revival model—OS.

	coef	exp(coef)	coef lower 95%	coef upper 95%	exp(coef) lower 95%	exp(coef) upper 95%	*p*
Neutrophils to lymphocytes ratio	0.200420791	1.221916822	0.095941091	0.304900491	1.100694221	1.356490013	0.000170083
Bone Met. At ICIs start (yes)	0.67122497	1.956632669	0.342883051	0.999566888	1.409003971	2.717104763	6.15657E-05
Antidiabetic drugs (yes)	0.005305941	1.005320043	-0.457606979	0.468218862	0.632796129	1.597146919	0.982076833
Bone Met. At ICIs start (no)	-0.660990147	0.516339829	-0.988399525	-0.333580768	0.372171866	0.716354038	7.59377E-05
Age (elder)	0.228350723	1.256525941	-0.243846995	0.700548441	0.783607521	2.014857436	0.343220287
Statins (no)	0.346658904	1.41433422	-0.020156753	0.713474562	0.980045036	2.041070779	0.063988106
Corticoids pre-ICIs (yes)	0.457398416	1.57995824	0.025961919	0.888834913	1.026301865	2.432294166	0.037718031
Corticoids pre-ICIs (no)	-0.405030256	0.666956631	-0.823401194	0.013340681	0.438936206	1.013430065	0.057766435
Histology (SCC)	-0.172903729	0.841218596	-0.564946012	0.219138553	0.568390832	1.245003763	0.387362963
PD-L1 (positive)	-0.310966092	0.732738721	-0.702426453	0.08049427	0.495381823	1.083822636	0.119483491
CNS Met. At ICIs start (no)	-0.282501953	0.75389517	-0.666374841	0.101370935	0.513566969	1.106687075	0.149192943
ECOG at ICIs start	0.415477937	1.515094688	0.240227326	0.590728549	1.271538171	1.805303187	3.37429E-06
Antidiabetic drugs (no)	-0.005305941	0.99470811	-0.468218862	0.457606979	0.626116476	1.580287795	0.982076833
Platelets to lymphocytes ratio	0.148411544	1.159990185	0.029313649	0.267509439	1.029747523	1.306705964	0.014591131
PD-L1 (negative)	0.074079733	1.076892665	-0.517780677	0.665940143	0.595841446	1.946319479	0.806210744
Histology (ADK)	0.10350635	1.109052835	-0.260208791	0.46722149	0.770890614	1.595554765	0.577002722
First ICIs line (2)	-0.076817259	0.926059067	-0.406860824	0.253226306	0.665736838	1.288174765	0.648261009
Histology (NOS)	0.282587875	1.326558342	-0.610579559	1.17575531	0.543036056	3.24058967	0.535185369
Liver Met. At ICIs start (no)	-0.547148343	0.578597421	-0.903685844	-0.190610842	0.405073866	0.826454146	0.002631522
Liver Met. At ICIs start (yes)	0.563004598	1.755940478	0.203789588	0.922219608	1.226040153	2.514866217	0.002127086
Statins (yes)	-0.346658904	0.707046458	-0.713474562	0.020156753	0.489938914	1.020361272	0.063988106
LDH before ICIs start	0.332774234	1.394832357	0.19832572	0.467222747	1.219359499	1.595556769	1.22768E-06
Age category (young)	-0.011125596	0.988936065	-0.395552272	0.373301081	0.673308087	1.4525216	0.954765992
CNS Met. At ICIs start (yes)	0.294184921	1.342032051	-0.093853368	0.68222321	0.910416249	1.978270958	0.137301649
First ICIs line (>2)	0.280706749	1.324065264	-0.087423949	0.648837448	0.916288553	1.913315208	0.135042293

Betablockers (no)	0.048313605	1.049499732	-0.435068734	0.531695944	0.64722018	1.701816047	0.844691148
Smoking (yes)	-0.108306661	0.897352371	-0.601811169	0.385197846	0.547818545	1.469905108	0.667092144
Sex (male)	0.181972584	1.199581306	-0.184561389	0.548506557	0.831468895	1.730666436	0.330523261
irAEs (yes)	-0.877177159	0.415955432	-1.292179737	-0.462174581	0.274671419	0.62991236	3.43225E-05
Smoking (packs/year)	-0.07534275	0.927425557	-0.242745656	0.092060156	0.784471014	1.096430777	0.377713131
Histology (LCNEC)	0.092467587	1.096877588	-1.052222808	1.237157982	0.349160769	3.445806491	0.874200686
Betablockers (yes)	-0.048313605	0.952834926	-0.531695944	0.435068734	0.587607575	1.545069253	0.844691148
First ICIs line (1)	-0.142316666	0.867346554	-0.487084596	0.202451264	0.614415053	1.224400411	0.41848464
Age (middle)	-0.0991774	0.905582043	-0.435125487	0.236770687	0.647183449	1.267150511	0.562849926
irAEs (no)	0.877177159	2.404103716	0.462174581	1.292179737	1.58752243	3.64071371	3.43225E-05
Smoking (no)	0.108306661	1.114389433	-0.385197846	0.601811169	0.680316025	1.825421955	0.667092144
Sex (female)	-0.181972584	0.833624195	-0.548506557	0.184561389	0.577812096	1.202690811	0.330523261
